# The Associations of Pulse Pressure and Mean Arterial Pressure on Physical Function in Older Americans

**DOI:** 10.3390/geriatrics8020040

**Published:** 2023-03-29

**Authors:** Abigail Pleiss, Donald Jurivich, Lindsey Dahl, Brenda McGrath, Daniela Kin, Ryan McGrath

**Affiliations:** 1 Department of Geriatrics, University of North Dakota, Grand Forks, ND 58202, USA; 2 OCHIN, Inc., Portland, OR 97228, USA; 3 Department of Health, Nutrition, and Exercise Sciences, North Dakota State University, Fargo, ND 58108, USA; 4 Fargo VA Healthcare System, Fargo, ND 58102, USA

**Keywords:** blood pressure, geriatrics, muscle weakness, walking speed

## Abstract

Background: We sought to examine the associations of pulse pressure (PP) and mean arterial pressure (MAP) on physical function in older Americans. Methods: Our analytic sample included 10,478 adults aged ≥65 years from the 2006–2016 Health and Retirement Study. Handgrip strength, gait speed, and standing balance were collected using relatively standard protocols. PP and MAP were calculated from blood pressure measurements. Results: Older Americans with any abnormality in PP had 1.15 (95% confidence interval (CI): 1.05–1.25) greater odds for slowness and 1.14 (CI: 1.05–1.24) greater odds for poorer standing balance. Persons with any abnormality in MAP had 0.90 (CI: 0.82–0.98) decreased odds for weakness and 1.10 (CI: 1.01–1.20) greater odds for poorer standing balance. Those with low PP had 1.19 (CI: 1.03–1.36) greater odds for slow gait speed, while persons with low MAP had 1.50 (CI: 1.09–2.05) greater odds for weakness and 1.45 (CI: 1.03–2.04) greater odds for slowness. Older Americans with high PP had 1.13 (CI: 1.03–1.25) greater odds for slowness and 1.21 (CI: 1.10–1.32) greater odds for poorer balance, whereas those with high MAP had 0.87 (CI: 0.80–0.95) decreased odds for weakness. Conclusions: Cardiovascular dysfunction, as observed by PP and MAP, may help to explain some of our findings.

## 1. Introduction

The proportion of older adults is rapidly increasing both in the United States and globally [1]. With this quickly growing older population, it is critical for geriatricians and related healthcare providers to focus efforts on the early identification of functional problems commonly observed in older adults. For example, mobility limitations are highly prevalent in older adults [2], and poor mobility is associated with health outcomes (e.g., falls) that shorten independence and longevity [2]. Measures of physical function, such as handgrip strength, gait speed, and standing balance, are prognostic indicators of mobility [3]. While these measures are robust, they are not always incorporated into clinical practice. Thus, linkage of measures for physical function to elements of routine clinical practice such as vital signs could provide insights about risk factors in older adults for functional decline. A connection between vital signs and functional changes could help identify early risk and expedited action plans for preserving mobility during aging.

Screenings and examinations are the leading services provided at office-based physician visits for older adults [4]. Blood pressure measurements are similarly commonplace during healthcare examinations and home health assessments [5,6]. Indeed, measuring blood pressure for hypertension is critical for detecting chronic morbidities such as cardiovascular disease [7]. Importantly, hypertension and forms of hypotension (e.g., orthostatic) are also associated with diminished physical functioning (e.g., gait speed) during aging [8,9], including well-controlled hypertensive older adults analyzed over a 14-year follow-up study period [8]. The association between hypertension and decreased physical function may also help to explain why certain blood pressure measurements, such as postural hypotension, are recommended in fall risk assessments in primary care settings [10]. For example, hypertension is linked to concurrent impairments in mobility [11], and having higher blood pressure from earlier ages into midlife is associated with progressively worsening gait [12]. Conversely, older adults that experienced intensive blood pressure lowering treatment did not observe changes in gait speed or transitions in mobility status [13]. Mechanisms related to vascular senescence, stiffness, and altered ventricular-vascular coupling may partially explain why pulse pressure is associated with poor mobility [14]. Mean arterial pressure abnormalities may affect cerebral blood flow, which may contribute to mobility status in older adults [15]. Accordingly, mean arterial pressure and pulse pressure may reveal additional insights into how these blood pressure markers could be linked to physical function.

Mean arterial pressure can inform a healthcare provider about overall cardiac output and the amount resistance in the peripheral vasculature [16], while pulse pressure can infer arterial stiffness [17]. Although abnormalities in mean arterial pressure and pulse pressure are risk factors for cardiovascular-related morbidities [18,19], changes in these values point to poor cerebral blood flow, which is associated with decreased physical function in community-dwelling older adults [14,15]. Given that mean arterial pressure and pulse pressure may serve as an indicator of the blood circulation and vasculature deficits that contribute to physical dysfunction, evaluating these factors may allow us to conduct more sensitive and comprehensive functional assessments in older adults. Thus, this investigation sought to examine the associations of mean arterial pressure and pulse pressure on physical function in older Americans.

## 2. Materials and Methods

### 2.1. Participants

We performed a secondary analysis of publicly available Health and Retirement Study (HRS) data for this investigation. Individual HRS data files were joined with the RAND HRS as needed. The HRS monitors economic and health characteristics in Americans as they age [20]. New cohorts of participants are included in the HRS every 6 years. While persons must be aged over 50 years to participate in the HRS, specific physical measures such as gait speed and standing balance are only ascertained from those aged ≥65 years [21]. Starting in the 2006 wave, the HRS included enhanced face-to-face interviews, wherein trained interviewers visited participant residences to collect physical measures [20]. These enhanced interviews alternated completion at each HRS wave such that the physical measures were collected in a random half sample, while the other half sample completed the core interviews, often over the telephone [22]. Interview response rates for each HRS wave are >80% [22].

The analytic sample included the last wave (i.e., cross-section) wherein 12,108 Americans aged at least 65 years participated during the 2006–2016 waves of the HRS with complete information for handgrip strength, gait speed, and standing balance. Complete information for the physical measurements was part of our analytic sample inclusion criteria because each physical measure had different eligibility criteria in the HRS [21]. While the HRS uses a panel design, the last wave wherein older Americans participated was selected to best represent the most recent measures recorded. The University’s Behavioral Sciences Committee Institutional Review Board approved HRS protocols. Participants provided written informed consent before study entry. Additional details about the HRS are available elsewhere [23].

### 2.2. Measures

#### 2.2.1. Blood Pressure

The HRS used an Omron HEM-780 Intellisense Automated blood pressure monitor with ComFit cuff (Omron; Kyoto, Japan) to measure blood pressure. To begin the blood pressure measurement, HRS interviewers pressed the start button on the monitor, and the cuff automatically inflated as appropriate, and then deflated whereby blood pressure (systolic and diastolic) and pulse were displayed. The blood pressure monitor was not visible to participants and interviewers were advised not to respond if participants asked about their blood pressure measurements while they were in progress. Interviewers recorded each measurement and waited approximately 60 s between measures. Three measures were collected. More details about how the HRS measured blood pressure and pulse are available elsewhere [21].

Pulse pressure was calculated by taking the mean difference of the three measures for systolic and diastolic blood pressure. Persons with pulse pressure < 40 mmHg were considered as having low pulse pressure [17], while those with pulse pressure > 60 mmHg were classified as having high pulse pressure [24,25]. Mean arterial pressure was determined with standard formulas from the three systolic and diastolic blood pressure measurements [26]. Participants with mean arterial pressure < 70 mmHg and >100 mmHg had low and high mean arterial pressure, respectively [27]. Pulse pressure and mean arterial pressure were calculated from the blood pressure measurements included in the HRS.

#### 2.2.2. Physical Function

Handgrip strength was measured with a Smedley spring-type handgrip dynamometer (Scandidact; Odder, Denmark). HRS interviewers explained the handgrip strength test protocols to participants and then fit the dynamometer to the hand size of each person. Starting on the self-reported non-dominant hand, participants squeezed the handgrip dynamometer with maximal effort, twice on each hand, alternating between hands. Additional details about handgrip strength testing in the HRS are available elsewhere [21]. The highest recorded handgrip strength was used for determining weakness. Men with handgrip strength < 35 kg and women with handgrip strength < 20 kg were weak [28].

A walking course was created in an unobstructed and ideally non-carpeted area of participant residences for gait speed testing. The starting and ending points of the measured walking course were identified by placing a piece of tape on the floor. HRS interviewers provided instructions to participants before testing. Each participant placed their toes at the start of the walking course. Interviewers told participants when to begin walking with a verbal que. Participants walked at a normal pace across the 2.5 m course, and interviewers stopped timing when the participant’s foot contacted the floor beyond the finish line. When the first walking trial was completed, interviewers asked participants to walk back to the other side of the course for the second trial using the same gait speed protocol. More details about the walking speed test protocols in the HRS are available elsewhere [21]. The mean of the two trials was used to calculate slow gait speed and persons with a gait speed < 0.8 m/second were slow [28].

HRS interviewers identified a flat and preferably uncarpeted area in participant residences for testing standing balance. The HRS standing balance protocols start with the semi-tandem (moderate-level) stance. In brief, participants that could hold the semi-tandem position for 10 consecutive seconds received a point for completing both the semi-tandem and side-by-side (lower-level) poses. Further, participants that successfully completed the semi-tandem balance test were then asked to complete the full tandem pose (advanced-level) with the same 10 s procedure. Those that held the full tandem pose for 3–9 s were given another point, while persons that were able to maintain the full tandem stance for all 10 s were awarded two points. Alternatively, persons that were unable to maintain the semi-tandem (moderate-level) pose for 10 s were asked to complete the side-by-side tandem pose (lower-level), and those that would maintain the side-by-side position for 10 consecutive seconds received a point. Additional information about the standing balance protocols in the HRS are available elsewhere [21]. Scores ranged from 0 to 4 with lower scores indicating poorer balance.

#### 2.2.3. Covariates

Age, sex, race, height, and weight were self-reported. Body mass index was calculated as weight in kilograms divided by height in meters squared and those with a body mass index of <18.5 kg per meters squared were underweight. Participants reported their health as “excellent”, “very good”, “good”, “fair”, or “poor”. Respondents told interviewers if a healthcare provider had diagnosed them with diabetes, cancer, hypertension, lung disease, a heart condition, stroke, emotional or psychiatric problems, and arthritis. Persons reporting at least two conditions had multimorbidity. Participants indicated if they were current cigarette smokers or if they had ever smoked more than 100 cigarettes in their lifetime (former smoker).

The 35-point adapted Telephone Interview of Cognitive Status was used to evaluate cognitive functioning [29]. This version of the Telephone Interview of Cognitive Status is well validated for screening cognitive function for population-based studies such as the HRS. Those with scores < 11 were considered as having a cognitive impairment. Persons indicating that they engage in moderate-to-vigorous physical activity at least “once a week” were classified as participating in moderate-to-vigorous physical activity [30]. Depressive symptomology was examined with the 8-item Center for the Epidemiologic Studies Depression scale. Scores ranged from 0 to 8, with higher scores indicating more depressive symptoms and those with scores > 2 were considered as depressed [31]. Missing and implausible covariates were excluded (*n* = 1630).

### 2.3. Statistical Analysis

All secondary analyses of HRS data were performed with SAS 9.4 software (SAS Institute; Cary, NC, USA). The descriptive characteristics of the participants were presented as the mean ± standard deviation or frequency (percentage) for continuous or categorical variables, respectively. The means and 95% confidence intervals (CI) were also shown by weakness (weak, not-weak), slowness (slow, not-slow) and balance status (failed semi-tandem, passed semi-tandem) for making descriptive comparisons. Separate logistic regression models analyzed the associations of having either low or high pulse pressure (reference: normal pulse pressure) on weakness and slow gait speed. An ordinal logistic regression model also analyzed the associations of having either low or high pulse pressure (reference: normal pulse pressure) on poorer standing balance. Likewise, individual logit models examined the associations of having either low or high mean arterial pressure (reference: normal mean arterial pressure) on weakness and slow gait speed. Another ordinal logit model was run to evaluate the associations of high or low mean arterial pressure (reference: normal mean arterial pressure) on poorer standing balance.

Distinct logistic regression models determined the associations of (1) low pulse pressure, and (2) high pulse pressure (reference: normal pulse pressure) on weakness and slowness. An ordinal logistic model similarly examined the associations between these same pulse pressure groups and poorer standing balance. Similarly, individual logistic models analyzed the associations of (1) low mean arterial pressure, and (2) high mean arterial pressure (reference: normal mean arterial pressure) on weakness and slowness. Another ordinal logistic model was conducted for examining the associations of the same mean arterial pressure groups on poorer standing balance. All models were adjusted for age, sex, race, underweight status, self-rated health, cognitive function, cigarette smoking status, depression status, moderate-to-vigorous physical activity participation, and multimorbidity.

As additional analyses, we performed the same logistic regression modeling procedures from our principal analyses by sex (male, female) and race (white, not-white) status. Moreover, separate linear regression models examined the associations of having low or high pulse pressure (reference: normal pulse pressure), and low or high mean arterial pressure (reference: normal mean arterial pressure) on handgrip strength, gait speed, and balance scores. Linear regression models also analyzed the associations of low mean arterial pressure, and high mean arterial pressure (reference: normal mean arterial pressure) on handgrip strength, gait speed, and balance scores, while series another linear models evaluated the associations of low pulse pressure, and high pulse pressure (reference: normal pulse pressure) on handgrip strength, gait speed, and balance scores. Further, tertiles were generated from low and high pulse pressure and mean arterial pressure. Individual logistic regression models analyzed the association of the low and high pulse pressure (reference: normal pulse pressure) and mean arterial pressure tertiles (reference: normal mean arterial pressure) on weakness and slowness. Another series of ordinal logit models likewise analyzed the associations between these same pulse pressure and mean arterial pressure tertile groups on poorer standing balance. The results from our additional analyses were presented as supplementary and minimally discussed because they were not principal to our investigation. An alpha level of 0.05 was used for all analyses.

## 3. Results

The descriptive characteristics of the 10,478 participants included from the HRS are presented in Table 1. Overall, the participants were aged 75.6 ± 7.0 years and were mostly female (56.2%). The means and 95% CI for the descriptive characteristics by physical function status are shown in Table 2. A greater proportion of participants with high pulse pressure were weak (38.2%; CI: 36.6, 39.7), slow (37.4%; CI: 36.2, 38.6), and failed the semi-tandem balance test (40.6%; CI: 37.2, 44.0) relative to persons who were not-weak (33.0%; CI: 31.8, 34.1), not-slow (31.3%; CI: 29.9, 32.6), and passed the moderate-level balance test (34.5%; CI: 33.5, 35.4). Moreover, a higher proportion of older Americans with low mean arterial pressure were weak (2.6%; CI: 2.1, 3.1) and slow (2.2%; CI: 1.8, 2.5) compared to their not-weak (1.4%; CI: 1.0, 1.6) and not-slow (1.4%; CI: 1.0, 1.7) peers.

Table 3 shows the results for the associations of the combined pulse pressure and mean arterial pressure groups on physical function. Persons with low or high pulse pressure had 1.15 (CI: 1.05, 1.25) greater odds for slow gait speed and 1.14 (CI: 1.05, 1.24) greater odds for poorer standing balance. Those with high or low mean arterial pressure had 1.10 (CI: 1.01, 1.20) greater odds for poorer standing balance, but interestingly 0.90 (CI: 0.82, 0.98) decreased odds for weakness. Table 4 presents the results for the associations between the individual pulse pressure and mean arterial pressure groups on physical function. Older Americans with low and high pulse pressure had 1.19 (CI: 1.03, 1.36) and 1.13 (CI: 1.03, 1.25) greater odds for slow gait speed, respectively. Further, persons with high pulse pressure had 1.21 (CI: 1.10, 1.32) greater odds for poorer standing balance. Those with low mean arterial pressure had 1.50 (CI: 1.09, 2.05) greater odds for weakness and 1.45 (CI: 1.03, 2.04) greater odds for slow gait speed, but persons with high pulse pressure interestingly had 0.87 (CI: 0.80, 0.95) decreased odds for weakness.

**Figure 1:**
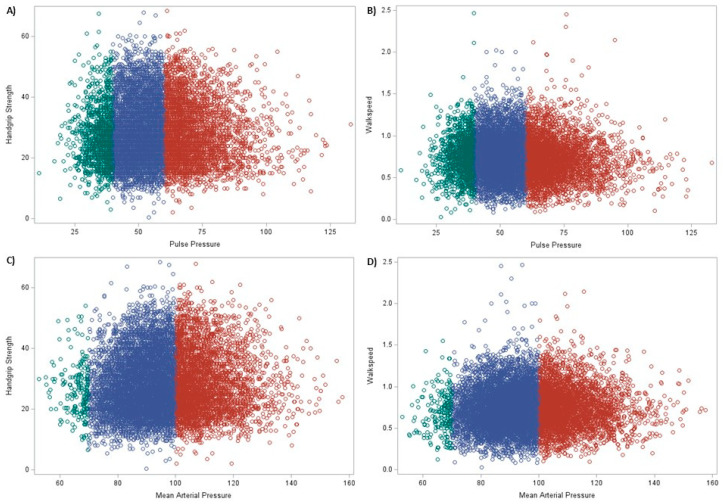
Scatter plots of mean arterial pressure and pulse pressure by handgrip strength and gait speed. Note: (**A**) = pulse pressure and handgrip strength, (**B**) = pulse pressure and gait speed, (**C**) = mean arterial pressure and handgrip strength, (**D**) = mean arterial pressure and gait speed, green = low pulse pressure or mean arterial pressure, blue = normal pulse pressure or mean arterial pressure, and red = high pulse pressure or mean arterial pressure.

The results for the associations of the combined pulse pressure and mean arterial pressure groups on physical function by sex and race status are in Table S1, while the results for the associations of the individual pulse pressure and mean arterial pressure groups on physical function by sex and race are in Table S2. Table S3 shows the results of the associations of the combined pulse pressure and mean arterial pressure groups on continuous measures of physical function, whereas Table S4 presents the results for the association of the individual pulse pressure and mean arterial pressure groups on continuous measures of physical function. Table S5 shows the associations of the tertile pulse pressure and mean arterial pressure groups on physical function. Scatter plots of mean arterial pressure and pulse pressure by handgrip strength and gait speed are in Figure 1.

## 4. Discussion

The principal results of this investigation suggest that pulse pressure and mean arterial pressure were differentially associated with weakness, slowness, and poorer balance in older Americans. Specifically, having any abnormality in pulse pressure was associated with 15% increased odds for slow gait speed and 14% increased odds for poorer standing balance. While having any abnormality in mean arterial pressure was associated with 10% increased odds for poorer standing balance, 10% decreased odds for weakness was observed. Persons with low pulse pressure had 19% increased odds for slow gait speed, while those with high pulse pressure had 13% increased odds for slow gait speed and 21% increased odds for poorer balance. Moreover, older Americans with low mean arterial pressure had 50% greater odds for weakness and 45% greater odds for slow gait speed, but persons with high mean arterial pressure had 13% decreased odds for weakness. The physiological processes that drive abnormal mean arterial pressure and pulse pressure may factor into physical function changes.

Previous studies have shown an association between higher blood pressure and elevated pulse pressure with declining in gait speed [8,14]. The mechanisms relating poor blood pressure control and impaired mobility could be related to vascular damage and white matter hyperintensities [11]. White matter hyperintensities are commonly observed on magnetic resonance imaging in older adults and are observed more frequently in persons with hypertension [8]. Such hyperintensities have been associated with stroke and small vessel disease [32], and may negatively impact the neural motor pathways linked to the executive function regions necessary for gait and other mobility tasks [33,34,35].

Additionally, endothelial cell dysfunction is associated with elevated pulse pressure [36]. The elevated pressures lead to pathological stretch in the vasculature, and thus, cerebral endothelial cells are stimulated to produce more reactive oxygen species and inflammatory cytokines [37]. These molecules, compounding with the excessive mechanical force, influence overall cellular function and cell death [38]. This inflammatory response also contributes to arterial stiffening [37], while endothelial cell apoptosis starts to break-down the blood–brain barrier [38]. Over time, endothelial cell dysfunction and blood–brain barrier breakdown result in cerebral microbleeds and hypoperfusion [38]. As the common cause hypothesis suggests, this vascular damage likely factors into a decline in both cognitive and sensorimotor functions [39], and in this case of our secondary analysis, slower gait speed.

Abnormalities in both mean arterial pressure and pulse pressure were similarly associated with worsening standing balance in our secondary analysis. Poor standing balance is a major predictor of fall risk, and elevated pulse pressures have previously been linked to an elevated risk of fall-related injury in older adults [40]. The SPRINT trial showed that persons with high pulse pressure were at greater risk for a serious adverse event including injurious falls [41,42]. Elevated pulse pressures may reflect increased arterial stiffness, whether due to the build-up of atherosclerotic plaques or natural changes with age. The inflexibility of the vasculature makes it difficult to maintain postural control after losing balance [16,43]. Additionally, the endothelial dysfunction observed with elevated pulse pressures may impair oxygen delivery to peripheral tissue [14]. When such pressures are low, persons are at risk of cerebral hypoperfusion, which has also been linked to postural instability [40].

Our analysis also showed an association between elevated mean arterial pressure and decreased odds for weakness. While this finding is more challenging to explain, it is possible that the mechanism relates to vascular resistance. A reduction in sympatholysis and restructuring of the microvascular network are both observed in older adults [44]. Moreover, aging is accompanied by worsening vasodilation function. Together, these mechanisms contribute to an overall increase in peripheral vascular resistance seen with increased age. To provide adequate perfusion to peripheral tissues despite the increased resistance, an increased compensatory arterial pressure needs to occur. The elevated arterial pressure is necessary to help prevent weakness and preserve normal muscular function [44]. Thus, higher mean arterial pressure is present in persons who do not have weakness, and those with low mean arterial pressure could be weak.

Our supplementary analyses revealed additional insights that couple with our principal findings. For example, our sex-stratified findings showed differential associations between pulse pressure and mean arterial pressure abnormalities on physical functioning. Although categorical variables such as weakness, slowness, and poorer balance present clinical utility and elevated interpretation, continuous variables provide increased granularity [45]. As such, the findings from our principal analyses pair with the supplementary findings wherein handgrip strength, gait speed, and balance scores were treated as continuous response variables. Moreover, the supplementary analyses whereby pulse pressure and mean arterial pressure abnormalities were categorized into tertiles showed that persons grouped in the tertiles furthest from the normal ranges might be at greater odds for poorer physical function.

The principal findings from our secondary analysis investigation suggest that abnormalities in pulse pressure and mean arterial pressure may generally contribute to the physical dysfunction that reduces independence and mobility. Blood pressure screenings and subsequent calculations of pulsatile pressure and mean arterial pressure may be useful additions to initial mobility assessments. These measurements are affordable and feasible to attain, and in most cases, standard blood pressure is already being recorded during patient encounters. Future research should aim to clarify the mechanisms underlying decreased physical function with abnormalities in both pulse pressure and mean arterial pressure with longitudinal designs.

Some limitations should be noted. Regarding limitations from the HRS, while there could be several physical function assessments that exist, the inclusion of handgrip strength, gait speed, and standing balance in our secondary analysis was based on data availability in the HRS. We chose not to control for blood pressure medications and related pharmacological treatments because specificity in medication type was unavailable. Population-based studies such as the HRS may experience challenges controlling for all factors that influence blood pressure. Regarding limitations from our secondary analysis, although the HRS utilizes a longitudinal-panel design, we analyzed the most recent wave in which participants had information for the physical measures included. This decision was chosen because the physical measures were completed concurrently in each biennial enhanced face-to-face interview with different inclusion criteria, and therefore, our ability to conduct longitudinal analyses was limited [46]. The cross-sectional design utilized in this secondary analysis creates limitations for the direction of the associations.

## 5. Conclusions

This investigation revealed that abnormal pulse pressure and mean arterial pressure were differentially associated with weakness, slowness, and poorer balance in older Americans. Our findings suggest pulse pressure and mean arterial pressure could be considered for physical function examinations and related comprehensive fall risk assessments. During clinic visits, mean arterial pressure and pulse pressure should be calculated and tracked alongside physical function exams. Additionally, abnormalities in these measures likely warrant a more extensive functional assessment. Pulse pressure and mean arterial pressure could be helpful markers for examining relevant declines in cardiovascular functioning that influence mobility-related tasks in older adults.

## Figures and Tables

**Table 1:** Descriptive Characteristics of the Participants.

Variable	Overall (*n* = 10,478)
Weakness (*n* (%))	3990 (38.1)
Slowness (*n* (%))	6261 (59.8)
Balance Score	3.4 ± 0.9
Age (years)	75.6 ± 7.0
Young Old (*n* (%))	5086 (48.5)
Female (*n* (%))	5884 (56.2)
Underweight (*n* (%))	193 (1.8)
Fair or Poor Self-Rated Health (*n* (%))	2920 (27.9)
Cognitive Impairment (*n* (%))	358 (3.4)
White Race (*n* (%))	8797 (84.0)
Current Smoker (*n* (%))	978 (9.3)
Depressed (*n* (%))	1977 (18.9)
MVPA Participation (*n* (%))	5439 (51.9)
Multimorbidity (*n* (%))	7895 (75.4)
Pulse Pressure (*n* (%))	
<40 mmHg	1211 (11.6)
40–60 mmHg	5600 (53.4)
>60 mmHg	3667 (35.0)
Mean Arterial Pressure (*n* (%))	
<70 mmHg	193 (1.9)
70–100 mmHg	6578 (62.7)
>100 mmHg	3707 (35.4)

Note: results are presented as the mean ± standard deviation or frequency (percentage) as indicated. MVPA = moderate-to-vigorous physical activity; Young Old = aged 65–74 years.

**Table 2:** Means and 95% Confidence Intervals for the Descriptive Characteristics by Physical Functioning Status.

Variable	Weak (*n* = 3990)	Not-Weak (*n* = 6488)	Slow (*n* = 6261)	Not-Slow (*n* = 4217)	Failed ST Balance Test (*n* = 812)	Passed ST Balance Test (*n* = 9666)
Age (years)	78.9 (78.7, 79.2)	73.6 (73.4, 73.7)	77.2 (77.0, 77.3)	73.3 (73.1, 73.5)	80.1 (79.5, 80.6)	75.2 (75.1, 75.4)
Young Old (%)	29.3 (27.9, 30.7)	60.3 (59.1, 61.5)	39.2 (38.0, 40.4)	62.3 (60.8, 63.7)	27.3 (24.2, 30.4)	50.3 (49.3, 51.3)
Female (%)	49.6 (48.0, 51.1)	60.1 (59.0, 61.3)	60.7 (59.5, 61.9)	49.4 (47.9, 50.9)	63.9 (60.6, 67.2)	55.5 (54.5, 56.5)
Underweight (%)	2.8 (2.3, 3.3)	1.2 (0.9, 1.5)	2.0 (1.7, 2.5)	1.4 (1.1, 1.8)	1.8 (0.9, 2.7)	1.8 (1.5, 2.1)
Fair or Poor SRH (%)	43.7 (33.2, 36.2)	23.6 (22.5, 24.6)	35.3 (34.1, 36.5)	16.7 (15.6, 17.8)	44.4 (41.0, 47.8)	26.4 (25.5, 27.3)
Cognitive Impairment (%)	5.3 (4.6, 6.0)	2.2 (1.8, 2.6)	4.8 (4.3, 5.4)	1.2 (0.9, 1.5)	8.1 (6.2, 10.0)	3.0 (2.6, 3.3)
White Race (%)	86.4 (85.4, 87.5)	82.4 (81.4, 83.3)	79.3 (78.3, 80.3)	90.7 (89.8, 91.6)	83.6 (81.0, 86.1)	83.9 (83.2, 84.7)
Current Smoker (%)	8.2 (7.4, 9.1)	9.9 (9.2, 10.7)	9.8 (9.1, 10.6)	8.5 (7.6, 9.3)	8.6 (6.6, 10.5)	9.3 (8.8, 9.9)
Depressed (%)	22.9 (21.6, 24.2)	16.3 (15.4, 17.2)	23.8 (22.8, 24.9)	11.4 (10.4, 12.3)	27.8 (24.7, 30.9)	18.1 (17.3, 18.8)
MVPA Participation (%)	45.6 (44.0, 47.1)	55.7 (54.5, 57.0)	43.9 (42.7, 45.2)	63.6 (62.2, 65.1)	37.4 (34.1, 40.7)	53.1 (52.1, 54.1)
Multimorbidity (%)	80.7 (79.5, 82.0)	72.0 (70.9, 73.1)	80.2 (79.2, 81.2)	68.0 (66.6, 69.5)	86.0 (83.7, 88.4)	74.4 (73.5, 75.3)
Blood Pressure Medication (%)	65.8 (64.3, 67.3)	61.4 (60.2, 62.6)	66.8 (65.7, 68.0)	57.5 (56.0, 59.0)	71.8 (68.7, 74.9)	62.3 (61.4, 63.3)
Pulse Pressure (%)						
<40 mmHg	10.5 (9.5, 11.4)	12.2 (11.4, 13.0)	11.6 (10.7, 12.3)	11.6 (10.6, 12.5)	9.7 (7.6, 11.7)	11.7 (11.0, 12.3)
40–60 mmHg	51.3 (49.7, 52.8)	54.8 (53.5, 55.9)	51.0 (49.7, 52.2)	57.1 (55.6, 58.6)	49.6 (46.1, 53.0)	53.8 (52.7, 54.7)
>60 mmHg	38.2 (36.6, 39.7)	33.0 (31.8, 34.1)	37.4 (36.2, 38.6)	31.3 (29.9, 32.6)	40.6 (37.2, 44.0)	34.5 (33.5, 35.4)
Mean Arterial Pressure (%)						
<70 mmHg	2.6 (2.1, 3.1)	1.4 (1.0, 1.6)	2.2 (1.8, 2.5)	1.4 (1.0, 1.7)	3.1 (1.8, 4.2)	1.8 (1.4, 2.0)
70–100 mmHg	64.2 (62.7, 65.7)	61.8 (60.6, 63.0)	62.7 (61.4, 63.8)	62.9 (61.4, 64.3)	61.4 (58.1, 64.8)	62.9 (61.9, 63.8)
>100 mmHg	33.1 (31.6, 34.5)	36.8 (35.6, 37.9)	35.1 (33.9, 36.2)	35.7 (34.2, 37.1)	35.4 (32.1, 38.7)	35.3 (34.4, 36.3)

Note: Use of blood pressure medications were self-reported. MVPA = moderate-to-vigorous physical activity, SRH = self-rated health, ST = semi-tandem; Young Old = aged 65–74 years.

**Table 3:** Associations of the Combined Pulse Pressure and Mean Arterial Pressure Groups on Physical Function.

Variable	Weakness			Slowness			Poorer Balance	
	*n*	Odds Ratio	95% CI	*n*	Odds Ratio	95% CI	Odds Ratio	95% CI
Pulse Pressure †								
<40 mmHg or >60 mmHg	1943	1.01	0.93, 1.10	3070	1.15	1.05, 1.25	1.14	1.05, 1.24
Mean Arterial Pressure ‡								
<70 mmHg or >100 mmHg	1426	0.90	0.82, 0.98	2337	0.99	0.91, 1.09	1.10	1.01, 1.20

† Reference: 40–60 mmHg. ‡ Reference: 70–100 mmHg. Note: Abnormal Pulse Pressure: *n* = 13 for 0 score, *n* = 396 for 1 score, *n* = 605 for 2 score, *n* = 599 for 3 score, *n* = 3265 for 4 score. Abnormal Mean Arterial Pressure: *n* = 12 for 0 score, *n* = 301 for 1 score, *n* = 456 for 2 score, *n* = 459 for 3 score, *n* = 2672 for 4 score. CI = confidence interval.

**Table 4:** Associations of the Individual Pulse Pressure and Mean Arterial Pressure Groups on Physical Function.

Variable	Weakness			Slowness			Poorer Balance	
	*n*	Odds Ratio	95% CI	*n*	Odds Ratio	95% CI	Odds Ratio	95% CI
Pulse Pressure †								
<40 mmHg	419	1.10	0.96, 1.27	723	1.19	1.03, 1.36	0.92	0.80, 1.07
>60 mmHg	1524	0.98	0.90, 1.08	2347	1.13	1.03, 1.25	1.21	1.10, 1.32
Mean Arterial Pressure ‡								
<70 mmHg	105	1.50	1.09, 2.05	137	1.45	1.03, 2.04	1.32	0.98, 1.77
>100 mmHg	1321	0.87	0.80, 0.95	2200	0.98	0.90, 1.07	1.09	0.99, 1.19

† Reference: 40–60 mmHg. ‡ Reference: 70–100 mmHg. Note: Low Pulse Pressure: *n* = 0 for 0 score, *n* = 79 for 1 score, *n* = 110 for 2 score, *n* = 114 for 3 score, *n* = 908 for 4 score; High Pulse Pressure: *n* = 13 for 0 score, *n* = 317 for 1 score, *n* = 495 for 2 score, *n* = 485 for 3 score, *n* = 2357 for 4 score; Low Mean Arterial Pressure: *n* = 0 for 0 score, *n* = 25 for 1 score, *n* = 23 for 2 score, *n* = 29 for 3 score, *n* = 116 for 4 score; High Mean Arterial Pressure: *n* = 12 for 0 score, *n* = 276 for 1 score, *n* = 433 for 2 score, *n* = 430 for 3 score, *n* = 2556 for 4 score. CI = confidence interval.

## Data Availability

Health and Retirement Study data are publicly available online: https://hrs.isr.umich.edu/data-products.

## Supplementary Materials

The following supporting information can be downloaded at: https://www.mdpi.com/article/10.3390/geriatrics8020040/s1, Table S1: Associations of the Combined Pulse Pressure and Mean Arterial Pressure Groups on Physical Function by Sex and Race Status; Table S2: Associations of the Individual Pulse Pressure and Mean Arterial Pressure Groups on Physical Function by Sex and Race Status; Table S3: Associations of the Combined Pulse Pressure and Mean Arterial Pressure Groups on Continuous Measures of Physical Function; Table S4: Associations of the Individual Pulse Pressure and Mean Arterial Pressure Groups on Continuous Measures of Physical Function; Table S5. Associations of the Tertile Pulse Pressure and Mean Arterial Pressure Groups on Physical Function.

## References

[1] Kanasi E., Ayilavarapu S., Jones J. (2016). The aging population: Demographics and the biology of aging. Periodontol. 2000.

[2] Freiberger E., Sieber C.C., Kob R. (2020). Mobility in older community-dwelling persons: A narrative review. Front. Physiol..

[3] Beaudart C., Rolland Y., Cruz-Jentoft A.J., Bauer J.M., Sieber C., Cooper C., Al-Daghri N., De Carvalho I.A., Bautmans I., Bernabei R. (2019). Assessment of muscle function and physical performance in daily clinical practice. Calcif. Tissue Int..

[4] Ashman J.J., Rui P., Okeyode T. (2016). Characteristics of Office-Based Physician Visits. https://www.cdc.gov/nchs/products/databriefs/db331.htm#:~:text=During%202016%2C%20the%20overall%20rate,than%20the%20rate%20for%20males.

[5] Sazlina S. (2015). Health screening for older people—What are the current recommendations?. Malays. Fam. Physician Off. J. Acad. Fam. Physicians Malays..

[6] Hwang K.O., Thomas E.J., Petersen L.A. (2018). Use of home blood pressure results for assessing the quality of care for hypertension. JAMA.

[7] Fuchs F.D., Whelton P.K. (2020). High blood pressure and cardiovascular disease. Hypertension.

[8] Rosano C., Longstreth W.T., Boudreau R., Taylor C.A., Du Y., Kuller L.H., Newman A.B. (2011). High blood pressure accelerates gait slowing in well-functioning older adults over 18-years of follow-up. J. Am. Geriatr. Soc..

[9] Briggs R., Donoghue O.A., Carey D., O’Connell M.D., Newman L., Kenny R.A. (2020). What is the relationship between orthostatic blood pressure and spatiotemporal gait in later life?. J. Am. Geriatr. Soc..

[10] Phelan E.A., Mahoney J.E., Voit J.C., Stevens J.A. (2015). Assessment and management of fall risk in primary care settings. Med. Clin..

[11] Hajjar I., Quach L., Yang F., Chaves P.H., Newman A.B., Mukamal K., Longstreth W., Inzitari M., Lipsitz L.A. (2011). Hypertension, white matter hyperintensities, and concurrent impairments in mobility, cognition, and mood: The Cardiovascular Health Study. Circulation.

[12] Mahinrad S., Kurian S., Garner C.R., Sedaghat S., Nemeth A.J., Moscufo N., Higgins J., Jr D.R.J., Hausdorff J.M., Lloyd-Jones D.M. (2020). Cumulative blood pressure exposure during young adulthood and mobility and cognitive function in midlife. Circulation.

[13] Odden M.C., Peralta C.A., Berlowitz D.R., Johnson K.C., Whittle J., Kitzman D.W., Beddhu S., Nord J.W., Papademetriou V., Williamson J.D. (2017). Intensive blood pressure control, gait speed, and mobility limitation: The Systolic Blood Pressure Intervention Trial. JAMA Intern. Med..

[14] Heffernan K.S., Manini T.M., Hsu F.C., Blair S.N., Nicklas B.J., Kritchevsky S.B., Fielding R.A. (2012). Relation of pulse pressure to long-distance gait speed in community-dwelling older adults: Findings from the LIFE-P study. PLoS ONE.

[15] Hussain S.M., Ernst M.E., Barker A.L., Margolis K.L., Reid C.M., Neumann J.T., Tonkin A.M., Phuong T.L.T., Beilin L.J., Pham T. (2022). Variation in Mean Arterial Pressure Increases Falls Risk in Elderly Physically Frail and Prefrail Individuals Treated with Antihypertensive Medication. Hypertension.

[16] Turusheva A., Frolova E., Kotovskaya Y., Petrosyan Y., Dumbadze R. (2020). Association between arterial stiffness, frailty and fall-related injuries in older adults. Vasc. Health Risk Manag..

[17] Homan T.D., Bordes S., Cichowski E. Physiology, Pulse Pressure. https://www.ncbi.nlm.nih.gov/books/NBK482408/.

[18] Liu M., Chen X., Zhang S., Lin Y., Xiong Z., Zhong X., Guo Y., Sun X., Zhou H., Xu X. (2021). Long-term visit-to-visit mean arterial pressure variability and the risk of heart failure and all-cause mortality. Front. Cardiovasc. Med..

[19] Selvaraj S., Steg P.G., Elbez Y., Sorbets E., Feldman L.J., Eagle K.A., Ohman E.M., Blacher J., Bhatt D.L., REACH Registry Investigators (2016). Pulse pressure and risk for cardiovascular events in patients with atherothrombosis: From the REACH registry. J. Am. Coll. Cardiol..

[20] Fisher G.G., Ryan L.H. (2018). Overview of the health and retirement study and introduction to the special issue. Work Aging Retire..

[21] Crimmins E., Guyer H., Langa K., Ofstedal M.B., Wallace R., Weir D. Documentation of Physical Measures, Anthropometrics and Blood Pressure in the Health and Retirement Study. https://hrs.isr.umich.edu/sites/default/files/biblio/dr-011.pdf.

[22] Sonnega A., Faul J.D., Ofstedal M.B., Langa K.M., Phillips J.W., Weir D.R. (2014). Cohort profile: The health and retirement study (HRS). Int. J. Epidemiol..

[23] HRS Data Book. https://hrs.isr.umich.edu/about/data-book.

[24] Mancusi C., Losi M.A., Izzo R., Canciello C., Carlino M.V., Albano G., De Luca N., Trimarco B., de Simone G. (2018). Higher pulse pressure and risk for cardiovascular events in patients with essential hypertension: The Campania Salute Network. Eur. J. Prev. Cardiol..

[25] Mancia G., Fagard R., Narkiewicz K., Redon J., Zanchetti A., Böhm M., Christiaens T., Cifkova R., De Backer G., Dominiczak A. (2014). 2013 ESH/ESC practice guidelines for the management of arterial hypertension: ESH-ESC the task force for the management of arterial hypertension of the European Society of Hypertension (ESH) and of the European Society of Cardiology (ESC). Blood Press..

[26] DeMers D., Wachs D. Physiology, Mean Arterial Pressure. https://www.ncbi.nlm.nih.gov/books/NBK538226/.

[27] Meng L., Yu W., Wang T., Zhang L., Heerdt P.M., Gelb A.W. (2018). Blood pressure targets in perioperative care: Provisional considerations based on a comprehensive literature review. Hypertension.

[28] Cawthon P.M., Manini T., Patel S.M., Newman A., Travison T., Kiel D.P., Santanasto A.J., Ensrud K.E., Xue Q.-L., Shardell M. (2020). Putative cut-points in sarcopenia components and incident adverse health outcomes: An SDOC analysis. J. Am. Geriatr. Soc..

[29] Plassman B.L., Newman T.T., Welsh K.A., Helms M. (1994). Properties of the Telephone Interview for Cognitive Status: Application in epidemiological and longitudinal studies. Neuropsychiatry Neuropsychol. Behav. Neurol..

[30] Feng X., Croteau K., Kolt G.S., Astell-Burt T. (2016). Does retirement mean more physical activity? A longitudinal study. BMC Public Health.

[31] Turvey C.L., Wallace R.B., Herzog R. (1999). A revised CES-D measure of depressive symptoms and a DSM-based measure of major depressive episodes in the elderly. Int. Psychogeriatr..

[32] Debette S., Markus H. (2010). The clinical importance of white matter hyperintensities on brain magnetic resonance imaging: Systematic review and meta-analysis. BMJ..

[33] Koo B.B., Bergethon P., Qiu W.Q., Scott T., Hussain M., Rosenberg I., Caplan L.R., Bhadelia R.A. (2012). Clinical prediction of fall risk and white matter abnormalities: A diffusion tensor imaging study. Arch. Neurol..

[34] Moscufo N., Guttmann C.R., Meier D., Csapo I., Hildenbrand P.G., Healy B.C., Schmidt J.A., Wolfson L. (2011). Brain regional lesion burden and impaired mobility in the elderly. Neurobiol. Aging..

[35] Srikanth V., Phan T.G., Chen J., Beare R., Stapleton J.M., Reutens D.C. (2010). The location of white matter lesions and gait—A voxel-based study. Ann. Neurol. Off. J. Am. Neurol. Assoc. Child. Neurol. Soc..

[36] McEniery C.M., Wallace S., Mackenzie I.S., McDonnell B., Yasmin, Newby D.E., Cockcroft J.R., Wilkinson I.B. (2006). Endothelial function is associated with pulse pressure, pulse wave velocity, and augmentation index in healthy humans. Hypertension.

[37] Jufri N.F., Mohamedali A., Avolio A., Baker M.S. (2015). Mechanical stretch: Physiological and pathological implications for human vascular endothelial cells. Vasc. Cell..

[38] Levin R.A., Carnegie M.H., Celermajer D.S. (2020). Pulse pressure: An emerging therapeutic target for dementia. Front. Neurosci..

[39] Christensen H., Mackinnon A.J., Korten A., Jorm A.F. (2001). The “common cause hypothesis” of cognitive aging: Evidence for not only a common factor but also specific associations of age with vision and grip strength in a cross-sectional analysis. Psychol. Aging..

[40] Welmer A.-K., Wang R., Rizzuto D., Ek S., Vetrano D.L., Qiu C. (2020). Associations of blood pressure with risk of injurious falls in old age vary by functional status: A cohort study. Exp. Gerontol..

[41] Krishnaswami A., Kim D.H., McCulloch C.E., Forman D.E., Maurer M.S., Alexander K.P., Rich M.W. (2018). Individual and joint effects of pulse pressure and blood pressure treatment intensity on serious adverse events in the SPRINT trial. Am. J. Med..

[42] Wright J.T., Whelton P.K., Reboussin D.M. (2016). A randomized trial of intensive versus standard blood-pressure control. N. Engl. J. Med..

[43] Peultier-Celli L., Lion A., Buatois S., Watfa G., Gueguen R., Benetos A., Perrin P.P. (2021). Relation of arterial stiffness with postural control in older people. Eur. Geriatr. Med..

[44] Taekema D.G., Maier A.B., Westendorp R.G., Craen A.J.M.D., De Craen A.J. (2011). Higher blood pressure is associated with higher handgrip strength in the oldest old. Am. J. Hypertens..

[45] Mayya S.S., Monteiro A.D., Ganapathy S. (2017). Types of biological variables. J. Thorac. Dis..

[46] McGrath R., Lang J.J., Ortega F.B., Chaput J.-P., Zhang K., Smith J., Vincent B., Piñero J.C., Garcia M.C., Tomkinson G.R. (2022). Handgrip strength asymmetry is associated with slow gait speed and poorer standing balance in older Americans. Arch. Gerontol. Geriatr..

