# Supplementary material for: The Associations of Pulse Pressure and Mean Arterial Pressure on Physical Function in Older Americans
Source: Geriatrics (Basel). 2023 Mar 29;8(2):40. doi: 10.3390/geriatrics8020040 (PMC10137340; doi:10.3390/geriatrics8020040)
Supplement: Supplementary file 1 [file geriatrics-08-00040-s001.zip › geriatrics-2242289-supplementary.pdf]

Table S1. Associations of the Combined Pulse Pressure and Mean Arterial Pressure Groups on Physical Function by Sex and Race Status.

Variable	Weakness		Slowness		Poorer Balance	
	Odds Ratio	95% CI	Odds Ratio	95% CI	Odds Ratio	95% CI
**Males (n=4,594)**						
Pulse Pressure†						
<40 mmHg or >60 mmHg	1.01	0.89, 1.15	1.21	1.06, 1.37	1.10	0.96, 1.26
Mean Arterial Pressure‡						
<70 mmHg or >100 mmHg	0.89	0.78, 1.01	1.08	0.95, 1.23	1.03	0.89, 1.18
**Females (n=5,884)**						
Pulse Pressure†						
<40 mmHg or >60 mmHg	0.99	0.89, 1.12	1.10	0.98, 1.24	1.16	1.04, 1.30
Mean Arterial Pressure‡						
<70 mmHg or >100 mmHg	0.90	0.80, 1.02	0.93	0.82, 1.05	1.15	1.03, 1.29
**White (n=8,797)**						
Pulse Pressure†						
<40 mmHg or >60 mmHg	1.03	0.94, 1.13	1.14	1.04, 1.25	1.13	1.03, 1.24
Mean Arterial Pressure‡						
<70 mmHg or >100 mmHg	0.91	0.83, 1.00	1.01	0.91, 1.11	1.11	1.01, 1.22
**Not-White (n=1,681)**						
Pulse Pressure†						
<40 mmHg or >60 mmHg	0.88	0.71, 1.10	1.24	0.98, 1.58	1.19	0.96, 1.48
Mean Arterial Pressure‡						
<70 mmHg or >100 mmHg	0.83	0.66, 1.04	0.97	0.76, 1.24	1.06	0.85, 1.32

†Reference: 40-60 mmHg. ‡Reference: 70-100 mmHg.

Note: CI=confidence interval.

Table S2. Associations of the Individual Pulse Pressure and Mean Arterial Pressure Groups on Physical Function by Sex and Race Status.

Variable	Weakness		Slowness		Poorer Balance	
	Odds Ratio	95% CI	Odds Ratio	95% CI	Odds Ratio	95% CI
**Males (n=4,594)**						
Pulse Pressure†						
<40 mmHg	1.16	0.92, 1.47	1.34	1.05, 1.70	1.02	0.79, 1.31
>60 mmHg	0.98	0.86, 1.12	1.18	1.03, 1.35	1.12	0.97, 1.29
Mean Arterial Pressure‡						
<70 mmHg	2.28	1.42, 3.64	1.86	1.15, 3.02	1.34	0.89, 2.01
>100 mmHg	0.84	0.74, 0.96	1.05	0.92, 1.20	1.00	0.97, 1.16
**Females (n=5,884)**						
Pulse Pressure†						
<40 mmHg	1.05	0.88, 1.25	1.13	0.96, 1.35	0.90	0.75, 1.08
>60 mmHg	0.98	0.86, 1.11	1.09	0.95, 1.24	1.28	1.13, 1.44
Mean Arterial Pressure‡						
<70 mmHg	0.98	0.61, 1.54	1.14	0.70, 1.85	1.30	0.85, 1.99
>100 mmHg	0.90	0.80, 1.01	0.92	0.81, 1.04	1.15	1.02, 1.29
**White (n=8,797)**						
Pulse Pressure†						
<40 mmHg	1.09	0.94, 1.27	1.16	1.01, 1.35	0.89	0.76, 1.05

>60 mmHg	1.01	0.91, 1.12	1.12	1.02, 1.25	1.21	1.09, 1.33
Mean Arterial Pressure‡						
<70 mmHg	1.38	0.99, 1.93	1.55	1.08, 2.21	1.39	1.03, 1.89
>100 mmHg	0.89	0.80, 0.98	0.98	0.89, 1.08	1.09	0.99, 1.21
**Not-White (n=1,681)**						
Pulse Pressure†						
<40 mmHg	1.10	0.76, 1.59	1.43	0.95, 2.14	1.10	0.76, 1.57
>60 mmHg	0.83	0.66, 1.05	1.19	0.92, 1.54	1.22	0.97, 1.54
Mean Arterial Pressure‡						
<70 mmHg	2.83	1.00, 8.06	0.71	0.24, 2.08	0.66	0.21, 2.08
>100 mmHg	0.80	0.64, 1.00	0.98	0.77, 1.25	1.07	0.86, 1.35

†Reference: 40-60 mmHg. ‡Reference: 70-100 mmHg.

Note: CI=confidence interval.

Table S3. Associations of the Combined Pulse Pressure and Mean Arterial Pressure Groups on Continuous Measures of Physical Function.

Variable	Weakness		Slowness		Poorer Balance	
	β	95% CI	β	95% CI	β	95% CI
Pulse Pressure†						
<40 mmHg or >60 mmHg (n=4,878)	-0.02	-0.27, 0.22	-0.01	-0.02, -0.01	-0.04	-0.08, -0.01
Mean Arterial Pressure‡						
<70 mmHg or >100 mmHg (n=3,900)	0.62	0.37, 0.88	0.01	-0.01, 0.01	-0.03	-0.07, 0.01

†Reference: 40-60 mmHg. ‡Reference: 70-100 mmHg.

Note: CI=confidence interval.

Table S4. Associations of the Individual Pulse Pressure and Mean Arterial Pressure Groups on Continuous Measures of Physical Function.

Variable	Weakness		Slowness		Poorer Balance	
	β	95% CI	β	95% CI	β	95% CI
Pulse Pressure†						
<40 mmHg (n=1,211)	-0.45	-0.85, -0.05	-0.01	-0.03, -0.01	0.03	-0.02, 0.09
>60 mmHg (n=3,667)	0.12	-0.14, 0.39	-0.01	-0.02, -0.01	-0.07	-0.11, -0.03
Mean Arterial Pressure‡						
<70 mmHg (n=193)	-1.45	-2.36, -0.53	-0.03	-0.06, -0.01	-0.11	-0.25, 0.01
>100 mmHg (n=3,707)	0.73	0.48, 0.99	0.01	-0.01, 0.01	-0.03	-0.06, 0.01

†Reference: 40-60 mmHg. ‡Reference: 70-100 mmHg.

Note: CI=confidence interval.

Table S5. Associations of the Tertile Pulse Pressure and Mean Arterial Pressure Groups on Physical Function.

Variable	Weakness		Slowness		Poorer Balance	
	Odds Ratio	95% CI	Odds Ratio	95% CI	Odds Ratio	95% CI
**Lower Tertiles**						
Pulse Pressure†						
Tertile 1 (37.6-39.9 mmHg)	1.20	0.96, 1.51	1.16	0.92, 1.45	0.93	0.73, 1.19
Tertile 2 (34.0-37.5 mmHg)	1.03	0.82, 1.30	1.12	0.89, 1.39	0.87	0.68, 1.11

Tertile 3 (≤33.9 mmHg)	1.08	0.85, 1.36	1.29	1.02, 1.62	0.99	0.78, 1.25
Mean Arterial Pressure‡						
Tertile 1 (68.3-69.9 mmHg)	1.41	0.82, 2.40	1.28	0.71, 2.29	1.12	0.68, 1.87
Tertile 2 (65.3-68.2 mmHg)	1.47	0.85, 2.54	1.49	0.84, 2.64	1.18	0.69, 2.00
Tertile 3 (≤65.2 mmHg)	1.60	0.93, 2.77	1.59	0.86, 2.92	1.65	1.02, 2.69
**Higher Tertiles**						
Pulse Pressure†						
Tertile 1 (60.1-65.6 mmHg)	0.88	0.76, 1.00	0.99	0.86, 1.14	1.17	1.02, 1.34
Tertile 2 (65.7-73.6 mmHg)	1.01	0.89, 1.16	1.17	1.02, 1.35	1.20	1.05, 1.38
Tertile 3 (≥73.7 mmHg)	1.07	0.93, 1.22	1.27	1.10, 1.46	1.25	1.09, 1.42
Mean Arterial Pressure‡						
Tertile 1 (100.1-105.0 mmHg)	0.88	0.76, 1.01	0.97	0.85, 1.11	0.98	0.85, 1.13
Tertile 2 (105.1-112.2 mmHg)	0.86	0.75, 0.99	0.92	0.81, 1.06	1.05	0.92, 1.20
Tertile 3 (≥112.3 mmHg)	0.87	0.76, 0.99	1.05	0.91, 1.20	1.23	1.08, 1.40

†Reference: 40-60 mmHg. ‡Reference: 70-100 mmHg.

Note: CI=confidence interval.
